# Upregulation of cell-surface mucin MUC15 in human nasal epithelial cells upon influenza A virus infection

**DOI:** 10.1186/s12879-019-4213-y

**Published:** 2019-07-15

**Authors:** Zhuang Gui Chen, Zhao Ni Wang, Yan Yan, Jing Liu, Ting Ting He, Kim Thye Thong, Yew Kwang Ong, Vincent T. K. Chow, Kai Sen Tan, De Yun Wang

**Affiliations:** 1 Department of Pediatrics, The Third Affiliated Hospital of Sun Yat-sen University, Guangzhou, Guangdong China; 2 Department of Otolaryngology, Yong Loo Lin School of Medicine, National University of Singapore, 1E Kent Ridge Road, Singapore, 119228 Singapore; 3 Center for Interventional Medicine, The Fifth Affiliated Hospital of Sun Yat-sen University, Zhuhai, Guangdong China; 4 Department of Microbiology and Immunology, Yong Loo Lin School of Medicine, National University of Singapore, Singapore, Singapore

**Keywords:** MUC15, H3N2, Mucin, Immunomodulation

## Abstract

**Background:**

Cell-surface mucins are expressed in apical epithelial cells of the respiratory tract, and contribute a crucial part of the innate immune system. Despite anti-inflammatory or antiviral functions being revealed for certain cell-surface mucins such as MUC1, the roles of other mucins are still poorly understood, especially in viral infections.

**Methods:**

To further identify mucins significant in influenza infection, we screened the expression of mucins in human nasal epithelial cells infected by H3N2 influenza A virus.

**Results:**

We found that the expression of MUC15 was significantly upregulated upon infection, and specific only to active infection. While MUC15 did not interact with virus particles or reduce viral replication directly, positive correlations were observed between MUC15 and inflammatory factors in response to viral infection. Given that the upregulation of MUC15 was only triggered late into infection when immune factors (including cytokines, chemokines, EGFR and phosphorylated ERK) started to peak and plateau, MUC15 may potentially serve an immunomodulatory function later during influenza viral infection.

**Conclusions:**

Our study revealed that MUC15 was one of the few cell-surface mucins induced during influenza infection. While MUC15 did not interact directly with influenza virus, we showed that its increase coincides with the peak of immune activation and thus MUC15 may serve an immunomodulatory role during influenza infection.

**Electronic supplementary material:**

The online version of this article (10.1186/s12879-019-4213-y) contains supplementary material, which is available to authorized users.

## Background

Airway epithelium is the central component of the defense against respiratory pathogens through the combined functions of physical barrier and the regulation of both innate and adaptive immunity [1]. In the healthy state, integrated cell-cell junctions, the airway mucus layer and the beating cilia act as the physical barrier by clearance of particulates such as pathogens and preventing them from entry into submucosa. The airway mucus is a viscoelastic gel with a complicated composition including antimicrobial substances, cytokines and antioxidant proteins [2] where mucins also play the role of the structural framework.

To date, 22 types of mucin proteins have been discovered in humans [3]. The mucins can be further divided into two groups according to their subcellular localization: secreted mucins and membrane-tethered mucins. Secreted mucins, such as MUC5AC and MUC5B, are large glycoproteins mainly produced by goblet cells and submucosal glands [4]. Conversely, membrane-tethered mucins, also known as cell-surface mucins, are encoded by *MUC1, MUC3A/B, MUC4, MUC11, MUC12, MUC13, MUC15, MUC16, MUC17, MUC18, MUC20, MUC21* or *MUC22*; and consist of transmembrane domains that anchor themselves to the plasma membrane, and some of them may shed their extracellular fragments into the airway tract cavity [3].

There are two distinctive mucus layers [5, 6]. The apical layer is rich in the two well-known secreted mucins, MUC5AC and MUC5B; and this layer is stickier so that particulates in the airway tract cavity could cling to it and then be trapped. The lower layer, also called periciliary layer (PCL), is “watery” or less viscoelastic, and thus allows cilia to beat with less resistance. In this layer, most membrane-tethered mucins such as MUC1, MUC4 and MUC16 localize on microvilli, cilia or goblet cells. These tethered mucins are found to trap smaller adenoassociated virus. Therefore, unlike their secreted counterparts, cell surface mucins likely function as a selective barrier rather than a non-specific one [6, 7].

Furthermore, cell-surface mucin may function as immunomodulatory factors during invasion of pathogens and allergens, working in concert with other components of the immune system to exert a suitable immune response. Among the cell surface mucins, MUC1 is the most studied cell-surface mucin and its anti-inflammatory role initiated by bacterial and viral infection has been well established. MUC1 is upregulated by respiratory virus-induced cytokines such as TNFα and IL8. However, MUC1 can then diminish the levels of these inflammatory factors by suppressing Toll-like receptor (TLR) pathway as a feedback loop [8–11]. Recently, it was reported that MUC1 defends against influenza virus by directly interacting with the viral particles and eliminating viral entry into respiratory epithelial cells [12]. These results implied that cell-surface mucins might regulate the immune response in airway epithelial cells during bacterial or viral infection so as to reduce inflammation’s harmful effect on the host.

However, despite the many studies on MUC1, the role of other cell-surface mucins in the airway during viral infection has not been well illustrated and demonstrated. Considering that MUC1 plays a role in microbial infection, it is therefore interesting to investigate the expression of other cell-surface mucins in an airway infection model. We have previously established a human nasal epithelial cells (hNECs) model for influenza infection and the study of airway host factors [13–15]. Using this hNECs model, we investigated the expression and potential functions of these mucins in influenza A virus infection, which can potentially help identify other mucins that are significant in influenza infection as targets for diagnostic or treatment purposes.

## Methods

### Cell culture

This study was approved by the National Healthcare Group Domain-Specific Review Board of Singapore (Ethics approval number: DSRB D/11/228; IRB 13–509). Nasal epithelial biopsies were obtained from adult patients with inferior turbinate or nasal polyps, who were scheduled for septoplasty or polypectomy in the Department of Otolaryngology of the National University Hospital (Singapore). These fresh nasal epithelial tissues were used to derive human nasal epithelial stem/progenitor cells (hNESPCs) and then differentiated into human nasal epithelial cells (hNECs) in air-liquid interface (ALI) culture system as described previously [13, 16, 17]. Briefly, primary cells were expanded with B-ALI™ complete growth medium (Lonza, Walkersville, MD) for about 1 week, and then transferred onto 12-well 0.4 μm Transwell inserts (Corning, Corning, NY, USA). 4 days after seeding, growth medium was discarded and 700 μl of PneumaCult™-ALI Medium with inducer supplements (STEMCELL Technologies Inc., Vancouver, British Columbia, Canada) was added to the basal chamber. The cells were cultured in ALI culture for 4 weeks, changing media every 2–3 days, until they were fully differentiated.

### Viral infection of hNECs

Fully differentiated hNECs were infected with human influenza A virus (IAV) H3N2 (Aichi/2/1968, American Type Culture Collection, Manassas, Va), strains of seasonal H1N1 (A/Singapore/G2–25.1/2014), H3N2 (A/Singapore/G2–26.1/2014) and B/Victoria lineage (B/Singapore/G2–14.1/2014) virus at a multiplicity of infection (MOI) of 0.1 and incubated at 35 °C with 5% CO_2_ for up to 72 h post infection (hpi), as described previously [13]. The cell lysate, supernatant and monocellular suspension were obtained at 8, 16, 24, 48, 72 hpi. Uninfected controls were cultured in the same media without virus and were harvested at either 48 or 72 hpi.

### Poly (I:C) and UV-inactivated virus treatment

Differentiated hNECs were also treated with 25 μg/mL poly (I:C) or same volume of media for 8, 24 and 48 h, and Ultraviolet light (UV) inactivated H3N2 virus or same media without virus for 24 and 48 h at the same MOI of 0.1.

### Plaque assay

The cell supernatant was collected at set time-points by adding 150 μl 1× dPBS (Lonza, Walkersville, Md) into the apical chamber and then incubated at 35 °C for 10 min. The cell supernatant was preserved at − 80 °C until viral titration through plaque assay as described previously [13]. MDCK cells were cultured in 24-well plates overnight to 95% confluency. 100 μL of serially diluted (10^-1^ to 10^-4^), TPCK-trypsin (1 μg/mL) activated virus samples were added and incubated for 1 h at 35 °C where plates were rocked at 15 min interval. After incubation, the inoculums were discarded and 1 mL of 1.2% Avicel overlay (FMC BioPolymer, Philadelphia, Pa) was overlaid onto each well, then incubated for 65 to 72 h at 35 °C with 5% CO_2_. At the end of incubation, Avicel overlay was then aspirated and cells were fixed in formaldehyde (4% in 1× PBS) for 1 h. Cells were then washed with 1× PBS, and 1% crystal violet was added for 15 min to stain cells. Plates were then rinsed with running water and air-dried. The PFU values were calculated from the plates as follows:

Number of plaques × Dilution factor = Number of PFU per 100 μL.

### MUC15 siRNA transfection and infection

Differentiated hNECs were transfected with 2 μM non-targeting siRNA (NT-siRNA) or MUC15 siRNA in *Accell* siRNA delivery medium (GE Healthcare Dharmacon, Inc.) for 3 times at − 48, − 24 and 6 hpi. hNECs were then harvested at 24 and 48 hpi for RT-qPCR analysis.

### MUC15 recombinant protein treatment

MDCK cells in 24-well (about 3 × 10^5^ cells/well) were treated with human MUC15 recombinant protein (rMUC15, 24.14 kDa, LifeSpan BioSciences, Inc. Seattle, WA, USA) before or after the viral infection. For the pretreatment, H3N2 virus (5 × 10^4^ PFU/30 μl) was incubated with 0, 2 or 30 ng rMUC15 in 30 μl for 1 h on ice, followed by plaque assay on MDCK cells using the pretreated virus. For the post treatment, MDCK cells were infected with H3N2 virus at an MOI of 0.1 for 1 h, where the inoculum was then removed and 500 μl EMEM medium containing 0, 2 or 30 ng of rMUC15 and 1 μg/mL trypsin-TPCK was added. After incubation at 35 °C for 48 h, the culture supernatant was collected for plaque assay.

### RNA extraction, cDNA synthesis and qRT-PCR

Total RNA isolation and cDNA synthesis were performed using mirVana miRNA isolation kit (Life Technologies) and Maxima First-Strand cDNA Synthesis Kit (Thermo Fisher Scientific) following the respective manufacturers’ protocols. SYBR green–based qPCR analyses were conducted using specific primers purchased from Sigma-Aldrich (St Louis, Mo) (see Additional file 1: Table S1). All samples were tested in triplicate by using the GoTaq-qPCR Master Mix kit (Promega, Madison, Wis), with thermal cycling parameters of 95 °C, 15 s; 60 °C, 60 s each cycle for 40 cycles, at a thermal changing rate of 1.6 °C/s using ViiA 7 PCR machine (Applied Biosystems, Carlsbad, CA). Mean cycle thresholds (Ct) values were analyzed, and the relative gene expression normalized to housekeeping gene *PGK1* was calculated using the 2^-ΔΔCt^ method.

### Western blotting

Protein lysate of hNECs was harvested at various time-points using RIPA lysis buffer (Thermo fisher) containing 1× protease and phosphatase inhibitor cocktail (Thermo fisher) following the manufacturer’s instruction. After incubation on ice for 20 min, the cell lysate was centrifuged at 13,200 rpm for 20 min at 4 °C and then the supernatant was collected. Protein concentration was determined using BioRad protein assay (Bio-Rad, Hercules, Calif) according to the manufacturer’s protocol and the absorbance at 595 nm was measured using a microplate reader (Synergy/HT, Biotek, Winooski, VT). 30 μg of each protein sample was loaded and run with 10% SDS-PAGE gel, and then transferred to PVDF membranes (Bio-Rad). Membranes was blocked with 5% w/v skim milk in 1 × TBST (TRIS-buffered saline and 0.1% v/v Tween-20) at room temperature for 1 h and then incubated in the primary antibody solution against MUC15 (1:500 dilution, Abcam, Cambridge, UK), epithelial growth factor receptor (EGFR) (1:2000 dilution, Abcam), phospho-ERK (1:1000 dilution, Cell Signaling Technology), ERK (1:1000 dilution, Cell Signaling Technology), hemagglutinin (HA) (1:1000 dilution, Abcam), matrix protein 1 (M1), neuraminidase (NA) (1:1000 dilution, Sino Biological) and GAPDH (1:10000 dilution, Cell Signaling Technology) overnight at 4 °C and incubated in the HRP-conjugated secondary anti-mouse IgG (1:5000 dilution, Cell Signaling Technology) or anti-rabbit IgG light chain (1:2000, Abcam) for 1 h at room temperature. The ECL substrate was added (Cell Signaling Technology) to the blots and the blots were developed with scientific imaging films in the dark room. GAPDH was used to normalize sample loading. Semi-quantitative analysis was performed on the western blot bands where intensities of the bands were measured using image J.

### Immunoprecipitation

30 μl of protein G-Sepharose beads (GE Healthcare, Singapore) were prepared by incubating in chilled lysis buffer containing 1 μg of rabbit anti-MUC15 (ab171304, Abcam) or rabbit anti-IgG isotype control antibodies (ab27478, Abcam) and rotating for 2 h at 4 °C. The beads were then washed thrice with ice-cold lysis buffer, followed by the addition of 10% BSA and 300 μg of cell lysates from H3N2-infected hNECs for 2 h at 4 °C. Antibody-bound beads were heated at 95 °C for 3 min and then centrifuged to obtain the immunoprecipitated proteins in the supernatant. The immunoprecipitated proteins were then analyzed using Western-blotting (10% SDS-PAGE gel) with 10 μg of total protein lysates used as input controls as described above.

### Cytospin and immunofluorescence staining

At the stipulated time-points, hNECs were dissociated from Transwells with 1× Trypsin/EDTA solution (Gibco, Carlsbad, Calif). After fixation in 4% formaldehyde at room temperature for 10 min, and dual 1 × dPBS wash, single-cell suspension via cytospin was prepared at a density of 1 × 10^4^ cells/100 μl using Shandon Cytospin 3 Cytocentrifuge (Thermo Fisher Scientific) at 500 rpm for 5 min with mild acceleration.

The infected hNECs on the cytospin slides were permeabilized using 0.1% Triton X-100 at room temperature for 10 min, and washed three times with milli Q water. Cells were then blocked using 10% goat serum (DAKO, Glostrup, Denmark) for 30 min at room temperature, and incubated with primary antibody against MUC15 (1:50 dilution, Abcam) and HA (1:250, Abcam) in 1% goat serum (DAKO) at 4 °C overnight. After incubation, cells were washed three times with 1 × TBS, and incubated with fluorescent secondary antibody for 1 h at room temperature, followed by another three times of washing. Cells were then mounted using ProLong AntiFade reagent with DAPI and covered with microslides. Cytospin slides were observed using an Olympus IX51 fluorescence microscope under 40 × objective lens or 100 × oil lens.

### Statistical analysis

Data were analyzed using GraphPad Prism 7 (GraphPad Software, La Jolla, Calif) software. For qPCR, the expression change of *MUC* and host immune response genes was presented as fold change by normalizing with uninfected control in each group. Viral titer, mRNA or protein level was expressed as median and interquartile. Statistical significance was analyzed with one-way ANOVA and nonparametric, paired, Friedman test. *P* values of less than .05 were considered significant. Cross-correlation between *MUC15* mRNA level and the host immune response (2^-ΔΔCt^), or protein level of MUC15 and EGFR (intensities of blot bands) was analyzed by using Spearman correlation test.

## Results

### MUC15 was the most consistently upregulated mucin during H3N2 infection among other mucins tested

**Fig. 1:**
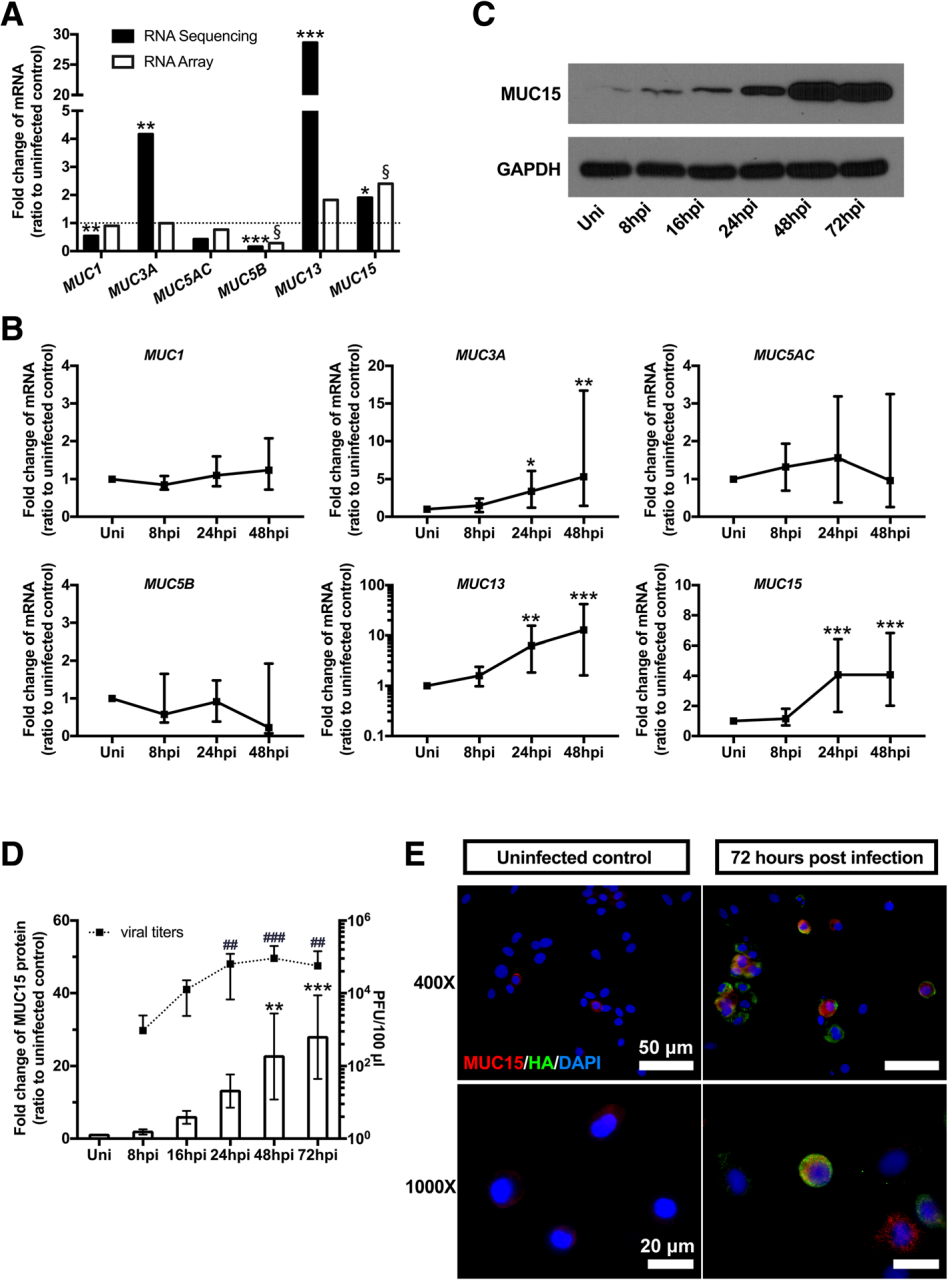
*MUC15* was upregulated after H3N2 infection in hNECs **a**: Fold changes of *MUC* mRNA expression in hNECs infected by H3N2 at 48 h post infection (hpi) with uninfected subjects (Uni) as baseline, detected by RNA sequencing (black column, *n* = 3) and mRNA array (white column, *n* = 5). **b**: Quantitative real-time RT-PCR analysis of *MUC* genes in hNECs infected with H3N2 with uninfected control subjects as baseline (*n* = 13), using *PGK1* as the internal control. Relative mRNA expression levels were calculated using 2^ ^(−ΔΔCt)^ method; the fold change was calculated compared against uninfected control subjects. **c**: Western blot analysis of MUC15 protein expression on hNECs infected with H3N2. Cells were harvested at various times as indicated. **d**: The western blot band intensities were measured using Image J, and graph is presented as the ratio of MUC15-to-GAPDH and then normalized to each uninfected control (*n* = 5) (left y axis and bars). Plaque assay show viral replication in hNECs infected by H3N2 (*n* = 8) (right y axis and spots). **e**: IF co-staining of MUC15 (red) and HA (green) on fully differentiated hNECs with or without H3N2 infection (Cell nuclei are stained in blue with DAPI, 49–6-Diamidino-2-phenylindole dihydrochloride). */§, **/^##^, ***/^###^, **** denotes *P* value of less than 0.05, 0.01, 0.001, < 0.0001 compared with uninfected control, respectively. Median values with 25th and 75th percentiles are indicated by error bar. Uni: uninfected control

To identify other *MUC* genes involved during IAV H3N2 infection in hNECs, Transcriptomes from RNA sequencing and mRNA microarray of infected hNECs were analyzed (from different donors for each assays). Among 19 tested *MUC* genes (*MUC1, 2, 3A, 3B, 4, 5 AC, 5B, 6, 7, 8, 12, 13, 15, 16, 17, 19, 20, 21, 22*), significant changes of mRNA levels were observed for *MUC1, 3A, 5B, 13, 15* by RNA sequencing; while *MUC3A, 5B, 15* were found to be significantly altered in mRNA array at 48 hpi compared with uninfected controls, with consistent directional changes in expression. However, *MUC5AC*, the most common airway *MUC* genes, did not show significant change of transcriptional expression (Fig. 1a), which is in line with our previous finding in influenza infection [13].

In order to confirm the transcriptional changes of these *MUC* genes after H3N2 infection, RT-PCR was carried out using cDNA from influenza virus infected cells and uninfected controls of 13 patients. The results showed that mRNA expressions of *MUC3A*, *13 and 15* were significantly upregulated at 24 and 48 hpi compared with the uninfected controls (Fig. 1b). Interestingly, the expression change of *MUC15* gene expression was the most significant and this increasing change was consistent across almost every hNEC sample from different donors (see Additional file 2: Table S2). In addition, we tested *MUC15* gene expression in other seasonal influenza virus strains (H1N1, H3N2 and B) and observed consistent upregulation across influenza strains (Additional file 3: Figure S1), a phenomenon not observed in *MUC13* and *MUC3A*. Therefore, we selected *MUC15* for further downstream investigations.

In addition, the protein expression of MUC15 was also upregulated upon H3N2 infection as demonstrated using western blotting (Fig. 1c and d). The immunofluorescence co-staining of MUC15 and influenza viral hemagglutinin (HA) showed that MUC15 was tethered on the cell plasma membrane, and that viral infection significantly increased MUC15 expression (Fig. 1e and Additional file 4: Figure S2). Co-staining of MUC15 and viral HA prompted us to further explore if there was any interaction between MUC15 and influenza surface protein in view of the membrane expression of MUC15.

### MUC15 up-regulation was induced only with active infection; and did not interfere with viral replication

**Fig. 2:**
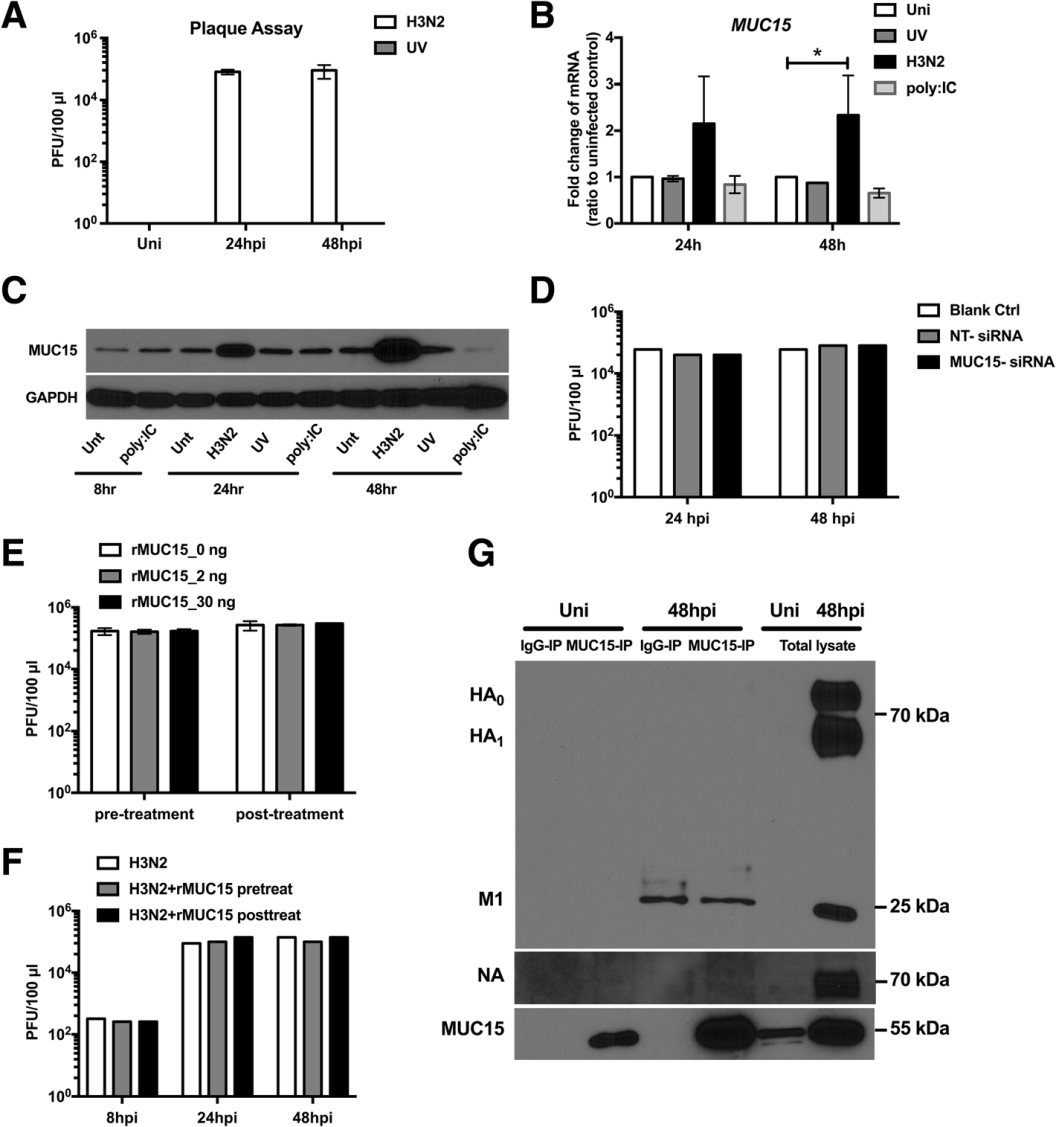
Active viral replication induced MUC15 upregulation but MUC15 did not interfere with viral replication **a**: Plaque assay showed viral replication in hNECs with or without H3N2 infection or UV-inactivated H3N2 (UV) (*n* = 2). **b**: Quantitative real-time RT-PCR analysis of *MUC15* mRNA expression in hNECs treated with or without H3N2, UV-inactivated H3N2 (UV) or 25 μg/mL poly (I:C) for 24 and 48 h with uninfected control subjects as baseline (*n* = 2), using *PGK1* as the internal control. Relative mRNA expression levels were calculated using 2^ ^(−ΔΔCt)^ method; the fold change was compared against uninfected control subjects. **c**: Western blot analysis of MUC15 protein expression on hNECs treated with or without H3N2, UV-inactivated H3N2 (UV) or 25 μg/mL poly (I:C). Cells were harvested at various times as indicated (*n* = 2). **d**: Viral replication in hNECs transfected with non-targeting siRNA (NT-siRNA) or MUC15-siRNA and normal hNECs (*n* = 1). **e-f**: Viral replication in MDCK cells (E, *n* = 3) and hNECs (F, *n* = 1) treated with 0, 2 or 30 ng recombinant MUC15 protein (rMUC15) before or after H3N2 infection. **g**: Immunoprecipitation with MUC15 protein followed by Western blotting for hemagglutinin (HA), matrix protein 1 (M1), neuraminidase (NA) and MUC15 (*n* = 2). Median values with 25th and 75th percentiles are indicated by error bar. * denotes *P* value of less than 0.05 compared with uninfected control. Uni: uninfected control; Unt: untreated control

IAV H3N2, UV-inactivated H3N2 and 25 μg/mL of poly (I:C) were used to treat fully differentiated hNECs to assess its upregulation in response to active, inactive and viral components. UV exposure successfully eliminated ability of viral replication as viral titers could be only detected in the H3N2 infected group but not in uninfected control or the UV-inactivated H3N2 treated group (Fig. 2a). Similarly, both transcriptional and translational expression of *MUC15* increased only in H3N2 infected hNECs, but not in UV-inactivated group and poly (I:C) group (Fig. 2b and c).

Although significant change of *MUC15* expression upon viral replication was elucidated, *MUC15* gene knockdown did not interfere with viral replication (Fig. 2d). Further experiments using human recombinant MUC15 protein (rMUC15) showed that MUC15 did not interact with or neutralize H3N2 virus. This is shown by the absence of significant changes in viral titer between rMUC15-treated group and the control, regardless of treatment before or after infection (Fig. 2e). The same experiment was carried out on hNECs in ALI culture and the result indicated that rMUC15 could not inhibit viral entry into hNECs or diminish viral replication (Fig. 2f). Furthermore, co-IP assay indicated no interaction between MUC15 and H3N2 viral proteins (HA, NA and M1; Fig. 2g).

### Expression of *MUC15* was positively correlated with inflammatory factors

**Fig. 3:**
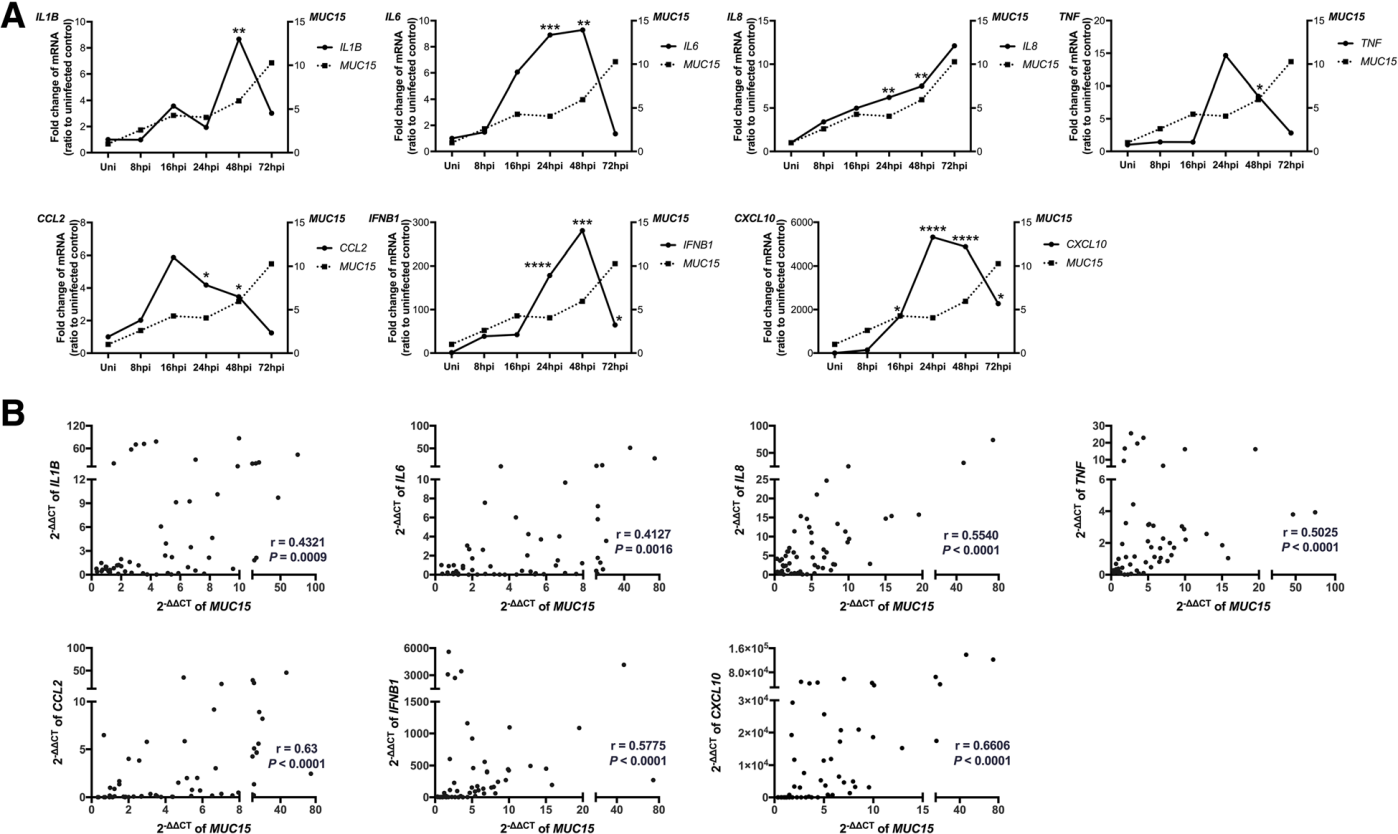
Expression of MUC15 was positively correlated with inflammatory factors **a**: Quantitative real-time RT-PCR analysis of *IL1B, IL6, IL8, TNF, CCL2, IFNB1, CXCL10,* (solid line, left y axis) and *MUC15* (dotted line, right y axis) mRNA expression in hNECs infected with H3N2 (*n* = 11), using *PGK1* as the internal control. Relative mRNA expression levels were calculated using 2^ ^(−ΔΔCt)^ method; the fold change was compared against uninfected control subjects. **b**: The correlations between mRNA level (2^ ^(−ΔΔCt)^) of *MUC15* versus *IL1B, IL6, IL8, TNF, CCL2, IFNB1, CXCL10* in hNECs infected with H3N2 were analyzed (*n* = 11). The timepoints post-infection that were included in the graphs are 8, 16, 24, 48 and 72 hpi. *, **, ***, **** denotes *P* value of less than 0.05, 0.01, 0.001, < 0.0001 compared with uninfected control, respectively. Uni: uninfected control

**Fig. 4:**
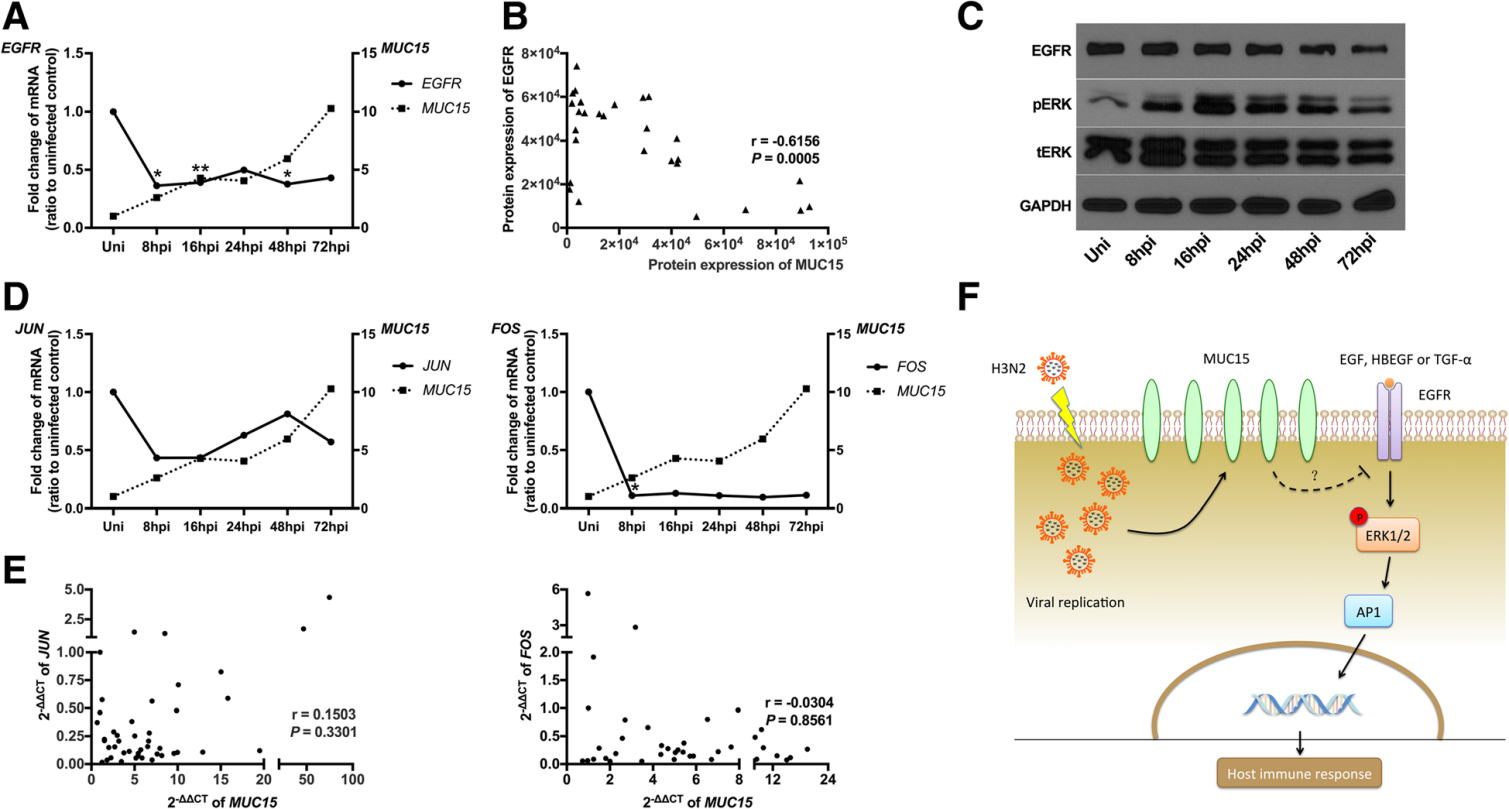
EGFR-pERK1/2-AP1 pathway was triggered by viral infection**. a**: Quantitative real-time RT-PCR analysis of *EGFR* (solid line, left y axis) and *MUC15* (dotted line, right y axis) mRNA expression in hNECs infected with H3N2 (*n* = 11), using *PGK1* as the internal control. Relative mRNA expression levels were calculated using 2^ ^(−ΔΔCt)^ method; the fold change was compared against uninfected control subjects. **b**: The correlations between protein level (blot band intensities) of MUC15 versus EGFR in hNECs with or without H3N2 infection were analyzed (*n* = 5). **c**: Western blot analysis of EGFR, pERK, total ERK and GAPDH protein expression on hNECs with or without H3N2infection. Cells were harvested at various times as indicated (*n* = 5). **d**: Quantitative real-time RT-PCR analysis of *JUN* and *FOS* mRNA expressions in hNECs with or without H3N2 infection (*n* = 11), using *PGK1* as the internal control. Relative mRNA expression levels were calculated using 2^ ^(−ΔΔCt)^ method; the fold change was compared against uninfected control subjects. **e**: The correlations between mRNA level (2^ ^(−ΔΔCt)^) of *MUC15* versus *JUN* and *FOS* in hNECs infected with H3N2 were analyzed (*n* = 11). **f**: The hypothesis of MUC15’s potential role in hNECs infected with H3N2: The increase of MUC15 expression triggered by H3N2 viral replication inhibits the activation of epithelial growth factor receptor (EGFR), which initiates the downstream ERK1/2-AP1 pathway. Thus, MUC15 may downregulate the host immune response at the later phase of viral infection. *, **, ***, **** denotes *P* value of less than 0.05, 0.01, 0.001, < 0.0001 compared with uninfected control, respectively. Uni: uninfected control

Since *MUC15* failed to hinder viral entry and viral replication of H3N2, we correlated its expression with the host immune responses induced after H3N2 infection of hNECs. Inflammatory cytokines and chemokines including *IL1B, IL6, IL8, TNF, IFNB1, CCL2* and *CXCL10* were increased both at mRNA levels (Fig. 3a) and protein levels as previously demonstrated [13]. Besides, there were positive correlations between mRNA levels of *MUC15* versus these inflammatory factors (Fig. 3b). Interestingly, the mRNA levels of most factors showed peak or plateauing trend at time-points coinciding with the increasing trend of *MUC15*. Additional MUC15 knockdown experiments in hNECs showed increase in immune responses (IFNβ and CXCL10) even at weak MUC15 reduction (Additional file 5: Figure S3). In addition, we detected reducing trends in expression of epithelial growth factor receptor (EGFR), ERK1/2 and pERK1/2, which are previously reported targets of MUC15 [18, 19]. In response to H3N2 infection, we observed that the protein expression of EGFR was downregulated during H3N2 infection in hNECs, which was negatively correlated with that of MUC15 (Fig. 4a and Fig. 4b). Besides, ERK1/2 and phosphorylated ERK1/2 were found to increase after H3N2 infection in hNECs and then decreased at 72 hpi, which coincided with increased expression of MUC15 (Fig. 4c). Downstream of EGFR-pERK/ERK signaling, the transcription factor *AP1* (*JUN* and *FOS*) was downregulated as well (Fig. 4d and e). Taken together, these findings suggested an interaction between MUC15 and immunomodulatory factors late into infection with H3N2 virus, and warrants further investigation to establish their exact immunomodulatory mechanisms.

## Discussion

The cell-surface mucins have been explored for their role in transduction of cellular signals, modulation of cell proliferation-differentiation and regulation of host immune response to microbial infections [3, 20]. Other than MUC1, levels of MUC4 and MUC16 were also significantly increased in inflamed conditions such as in COPD patients [21, 22]. With respect to this evidence, cell-surface mucins may play an important role in modulation of infection and inflammation. Acute excessive inflammation will impact the host when unregulated, from epithelium injury to systemic inflammatory response syndrome (SIRS), organ failure and even death. Besides, prolonged pathological inflammation can also result in chronic inflammatory diseases such as asthma and chronic obstructive pulmonary disease (COPD). Among these cell surface mucins, MUC1 is implicated in the pathogenesis of influenza infection [12]. Therefore, it is of great clinical significance to further identify if there are other cell-surface mucins involved in the pathogenesis of influenza infection, i.e. for their utility as markers or targets for influenza management.

In this study, we found that *MUC15* mRNA and protein expression was upregulated after IAV H1N1, H3N2 and influenza B infection in human nasal epithelial cells. MUC15 is a newly reported glycosylated protein with structural hallmarks of other membrane-tethered mucins, with an extracellular region with several potential glycosylation sites, a putative transmembrane domain, and a short cytoplasmic C-terminal [23]. The few studies on MUC15 focused on its association with genesis of carcinoma [18, 19, 24–26]. Although mRNA level of *MUC15* can be induced by respiratory syncytial virus (RSV) and human metapneumovirus (hMPV) infection in human epithelial cells [27], *MUC15* expression and function upon influenza virus infection are yet unclear. In addition, MUC1 is the first cell-surface mucin with anti-influenza function [12], and it was reported that MUC1 and MUC15 in the milk-fat globule membrane milk can limit bacterial and viral infections of the gastrointestinal tract [28–30]. Therefore, in view of its consistent elevation across different hNEC samples and influenza strains, we assessed the effects of MUC15 changes following influenza infection. Unlike MUC1 however, increase of this cell-surface mucin did not interfere with viral entry into epithelial cells by directly interacting with virus particles. Correlations of *MUC15* mRNA levels versus many immunological factors responding to influenza infection, have been revealed, and may be important for future studies to elucidate MUC15 immunomodulatory functions. Besides, we also detected two main cellular signaling factors – EGFR and phosphorylated ERK1/2 (found as MUC15 targets), which could be stimulated in response to viral infection, and may induce downstream reactions such as anti-viral cytokine production. We also observed that expression of EGFR and phosphorylation of ERK1/2 were reduced at late phase of viral infection (such as 48 hpi, 72 hpi), in response to MUC15 increase. In this respect, MUC15 may play an immunomodulatory role in human nasal epithelial cells infected by H3N2 through regulation of the EGFR-pERK1/2-AP1 pathway (Fig. 4f).

Wang et al. revealed that MUC15 could bind EGFR at the cell membrane to block the dimerization and activation of EGFR [19]. Thus, MUC15 could potentially exert its immunomodulatory functions via inhibition EGFR and its downstream pathway. On the other hand, MAPK pathway is a crucial signaling pathway upon viral infection. Ligands such as EGF, HBEGF, and TGF-α would stimulate EGFR to activate ERK1/2. Activator protein (AP1) is a heterodimer composed of c-Fos and c-Jun, whose transcriptional activity would be enhanced by phosphorylation of ERK1/2, and then promote the expression of IFN-β and anti-viral cytokines [31–33]. If MAPK pathway activation is reduced, anti-viral inflammation could be also suppressed, preventing over-inflammation to mitigate inflammatory damage to the host. In our study with hNECs, no direct interaction between MUC15 and EGFR was observed (data not shown); while treatment of rMUC15 protein also did not significantly reduce EGFR expression (data not shown). However, we observed decreased levels of EGFR, ERK and pERK in the late phase of infection, which coincided with strong induction of MUC15 in the nasal epithelium, indicating its potential role in immune-modulation, possibly via other indirect means.

Airway epithelium is a component of the innate immune system and its abnormal response to the pathogens or allergens may result in the genesis of chronic airway diseases. In recent years, some researchers have found that there was a strong link between early acute viral infection and subsequent chronic airway diseases [34, 35]. Furthermore, acute viral infection is also a common risk factor for the exacerbation of chronic airway diseases [35, 36]. Virus-derived inflammation clearly plays a central role in these processes. For instance, interleukin-1β (IL-1β) and interleukin-17A (IL-17A) may mediate neutrophilic inflammation in influenza-initiated exacerbation of chronic lung inflammation [37]. As a counterpart of MUC1, MUC15 was upregulated by several respiratory viruses, including influenza, as showed in our results, respiratory syncytial virus (RSV) and human metapneumovirus (hMPV). This increased expression occurred at the late phase of infection (48 or 72 hpi) [27], while most of virus-induced cytokines and chemokines peaked and plateaued. Therefore, we suspect that the increased MUC15 level may be in response to the activation of these factors and may play a role in their regulation.

Another interesting finding was that the upregulation of MUC15 expression only occurred during active infection of the nasal epithelial cells, rather than by UV-inactivated virus or viral mimics like poly (I:C). This indicated that increased MUC15 might be induced by the virus as a defense mechanism against host inflammatory responses. Future work can therefore focus on the interaction between the influenza NS1 protein and MUC15, and as a possible diagnosticbiomarker for severe influenza infections.

## Limitations

The main limitation in this study is that we could not ascertain the anti-inflammatory role of MUC15 during viral infection through the *MUC15* knockdown experiments, because the hNECs derived from nasal biopsies were poorly transfected. Attempts at siRNA transfection on hNECs yielded MUC15 knockdown in the absence of infection, but was weaker during infection due to the high levels of MUC15 expression and activation, albeit the increase of IFNβ and CXCL10 was observed. On the other hand, airway epithelial cell lines such as A549 cells expressed lower levels of MUC15 compared to hNECs and were thus not used for knockdown experiments (data not shown). Hence, in view of the interesting finding of MUC15 induction in hNECs, future studies on mechanistic investigation of MUC15 could include gene editing to completely knockout MUC15 to observe its loss of function effect following influenza infection, particularly the effects on innate immune responses. Gene editing studies on cell systems such as hNECs have been recently successfully explored, and can potentially be implemented for studies of cell surface mucins [38].

### Conclusion

The cell-surface mucin MUC15 was upregulated in hNECs after IAV H3N2 infection. Despite the lack of direct interaction with the virus to inhibit viral entry or viral replication, MUC15 showed a positive correlation with inflammatory factors at the level of mRNA expression. Therefore, it is possible that MUC15 may play an immunomodulatory role during H3N2 infection in the regulation of inflammatory responses. Nevertheless, in view of the strong induction of MUC15 following influenza infection of multiple strains, MUC15 may be exploited as a novel biomarker of influenza infection, and its functions during infection should be fully explored in the future.

## Additional files

Additional file 1: **Table S1.** Sequence of Primers for Real-Time Polymerase Chain Reaction. (DOCX 15 kb)

Additional file 2: **Table S2.** Fold change of *MUC15* mRNA expression. (DOCX 14 kb)

Additional file 3: **Figure S1.** Expression of *MUC15*, *MUC13* and *MUC3A* in seasonal influenza H1N1, H3N2 and B infection. Quantitative real-time RT-PCR analysis of *MUC15*, *MUC13* and *MUC3A* gene mRNA expression in hNECs infected with seasonal influenza H1N1, H3N2, and B, with uninfected control subjects as baseline (*n* = 4) and using *PGK1* as the internal control. Relative mRNA expression levels were calculated using 2^ ^(−ΔΔCt)^ method; the fold change was calculated as fold increases from uninfected control subjects. * and ** denotes *P* value of less than 0.05 and 0.01 compared with uninfected control, respectively. (PDF 37 kb)

Additional file 4: **Figure S2.** Mean fluorescence intensity (MFI) of MUC15 in infected hNECs. Five different images were captured from every slide and the mean immunofluorescence intensities (MFI) of MUC15 were measured using Image J (*n* = 5). Data was then normalized to each uninfected control. *, **, ***, **** denotes *P* value of less than 0.05, 0.01, 0.001, < 0.0001 compared with uninfected control, respectively. Median values with 25th and 75th percentiles are indicated by error bar. Uni: uninfected control. (PDF 21 kb)

Additional file 5: **Figure S3.** Expression of *MUC15*, *IFNβ* and *CXCL10* in MUC15 siRNA knockdown in hNECs. siRNA knockdown of *MUC15* in hNECs (*n* = 1). siRNA knockdown of *MUC15* was achieved stronger in non-infected hNECs (both 24 and 48 hpi). Conversely, the knockdown was greatly reduced in infected hNECs when *MUC15* is highly induced; and the knockdown is only slightly achieved at 24 hpi (grey bars). Quantitative real-time RT-PCR analysis of *MUC15*, *IFNβ* and *CXCL10* was performed using *PGK1* as internal control. Relative mRNA expression levels were calculated using 2^ ^(−ΔΔCt)^ method. Hpi: hours post infection; NT: non-targeting control; M15: MUC15 siRNA. (PDF 41 kb)

## Data Availability

The datasets used and/or analyzed during the current study available from the corresponding author on reasonable request.

## Abbreviations

ALI: Air-liquid interface
AP1: Activator protein 1
COPD: Chronic obstructive pulmonary disease
EGFR: Epithelial growth factor receptor
HA: Hemagglutinin
hMPV: human metapneumovirus
hNECs: human nasal epithelial cells
hNESPCs: human nasal epithelial stem/progenitor cells
hpi: hours post infection
IAV: Influenza A virus
IL-17A: Interleukin-17A
IL-1β: Interleukin-1β
M1: Matrix protein 1
MOI: Multiplicity of infection
NA: Neuraminidase
PCL: Periciliary layer
PGK1: Phosphoglycerate kinase 1
rMUC15: human MUC15 recombinant protein
RSV: Respiratory syncytial virus
SIRS: Systemic inflammatory response syndrome
TLR: Toll-like receptor
UV: Ultraviolet light
