# Supplementary material for: Upregulation of cell-surface mucin MUC15 in human nasal epithelial cells upon influenza A virus infection
Source: BMC Infect Dis. 2019 Jul 15;19:622. doi: 10.1186/s12879-019-4213-y (PMC6631914; doi:10.1186/s12879-019-4213-y)
Supplement: Supplementary file 1 — Table S1. Sequence of Primers for Real-Time Polymerase Chain Reaction. (DOCX 15 kb) [file 12879_2019_4213_MOESM1_ESM.docx]

**Supplementary Table 1 Sequence of Primers for Real-Time Polymerase Chain Reaction**

| **Primer** | **Sequence (5’ to 3’)** | | **Primer** | **Sequence (5’ to 3’)** |
| --- | --- | --- | --- | --- |
| AREG forward primer | AAAGAAAGAAAAAGGGAGGC | | IL8 forward primer | GTTTTTGAAGAGGGCTGAG |
| AREG reverse primer | CATTTGCATGTTACTGCTTC | | IL8 reverse primer | TTTGCTTGAAGTTTCACTGG |
| CCL2 forward primer | AGACTAACCCAGAAACATCC | | JUN forward primer | AAAGGATAGTGCGATGTTTC |
| CCL2 reverse primer | ATTGATTGCATCTGGCTG | | JUN reverse primer | TAAAATCTGCCACCAATTCC |
| CXCL10 forward primer | AAAGCAGTTAGCAAGGAAAG | | MUC1 forward primer | GCCTCTCCAATATTAAGTTCAG |
| CXCL10 reverse primer | TCATTGGTCACCTTTTAGTG | | MUC1 reverse primer | AGATCGTCAGGTTATATCGAG |
| EGFR forward primer | AGTGCCTGAATACATAAACC | | MUC13 forward primer | ATTTCTAATGCCTTGTCCTC |
| EGFR reverse primer | GTAGTGTGGGTCTCTGC | | MUC13 reverse primer | TTTCACCATCTAACAAAGGC |
| EGR1 forward primer | GCAGAGTCTTTTCCTGAC | | MUC15 forward primer | AGTTCTGCGATTAGACAATG |
| EGR1 reverse primer | TTGGTCATGCTCCACTAGG | | MUC15 reverse primer | ATACAGAAGTACGAAGTGGAG |
| FOS forward primer | CAGTTATCTCCAGAAGAAGAAG | | MUC3A forward primer | TCGTTGTGAAAACCACATAG |
| FOS reverse primer | CTTCTAGTTGGTCTGTCTCC | | MUC3A reverse primer | GAGAAAATAAGAACCAGGGC |
| HBEGF forward primer | GCTTATATACCTATGACCACAC | | MUC5AC forward primer | AATGGTGGAGATTTTGACAC |
| HBEGF reverse primer | GTACCTAAACATGAGAAGCC | | MUC5AC reverse primer | TTCTTGTTCAGGCAAATCAG |
| IFNB1 forward primer | ATTCTAACTGCAACCTTTCG | | MUC5B forward primer | ACAAGTCCATGGATATCGTC |
| IFNB1 reverse primer | GTTGTAGCTCATGGAAAGAG | | MUC5B reverse primer | ATTTGGTCAAACAGGATCAG |
| IL1B forward primer | CTAAACAGATGAAGTGCTCC | | PGK1 forward primer | CTCAACAACATGGAGATTGG |
| IL1B reverse primer | GGTCATTCTCCTGGAAGG | | PGK1 reverse primer | CTTTGGACATTAGGTCTTTGAC |
| IL6 forward primer | GCAGAAAAAGGCAAAGAATC | TNF forward primer | | AGGCAGTCAGATCATCTTC |
| IL6 reverse primer | CTACATTTGCCGAAGAGC | TNF reverse primer | | TTATCTCTCAGCTCCACG |
