# Supplementary material for: Upregulation of cell-surface mucin MUC15 in human nasal epithelial cells upon influenza A virus infection
Source: BMC Infect Dis. 2019 Jul 15;19:622. doi: 10.1186/s12879-019-4213-y (PMC6631914; doi:10.1186/s12879-019-4213-y)
Supplement: Supplementary file 2 — Table S2. Fold change of MUC15 mRNA expression. (DOCX 14 kb) [file 12879_2019_4213_MOESM2_ESM.docx]

**Supplementary Table 2 Fold change of MUC15 mRNA expression of MUC15**

| **Sample** | **Fold change of MUC15 mRNA expression*** | | |
| --- | --- | --- | --- |
|  | **8 hpi** | **24 hpi** | **48 hpi** |
| Patient 1 | 1.37 | 1.99 | 4.71 |
| Patient 2 | 1.85 | 10.19 | 17.32 |
| Patient 3 | 0.08 | 0.75 | 0.23 |
| Patient 4 | 0.64 | 0.86 | 3.06 |
| Patient 5 | 0.65 | 1.47 | 1.73 |
| Patient 6 | 0.81 | 4.32 | 3.42 |
| Patient 7 | 1.04 | 4.08 | 2.86 |
| Patient 8 | 0.94 | 1.33 | 1.25 |
| Patient 9 | 0.68 | 2.87 | 1.66 |
| Patient 10 | 1.52 | 9.62 | 3.92 |
| Patient 11 | 1.71 | 4.06 | 6.14 |
| Patient 12 | 3.52 | 5.26 | 7.06 |
| Patient 13 | 1.13 | 4.61 | 4.23 |

*Fold change of MUC15 mRNA expression reference to mock of each sample. Fold change larger than 1.00 means the mRNA expression increased.
