# Supplementary figures and images for: Upregulation of cell-surface mucin MUC15 in human nasal epithelial cells upon influenza A virus infection
Source: BMC Infect Dis. 2019 Jul 15;19:622. doi: 10.1186/s12879-019-4213-y (PMC6631914; doi:10.1186/s12879-019-4213-y)

*MUC15*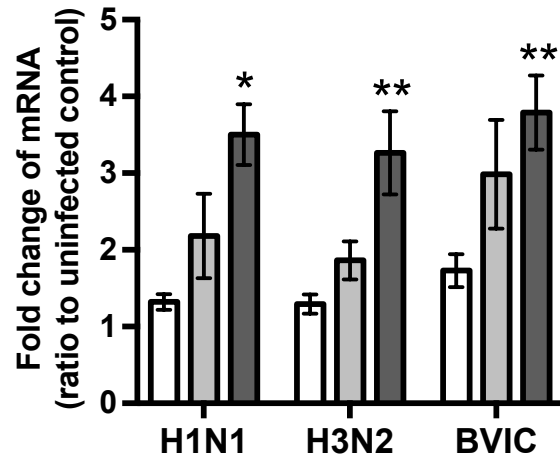*MUC13*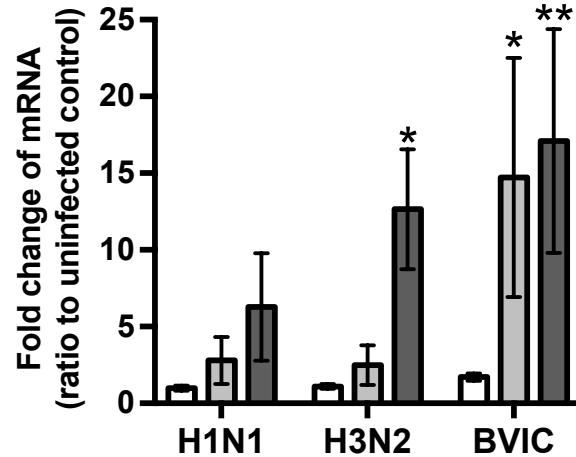*MUC3A*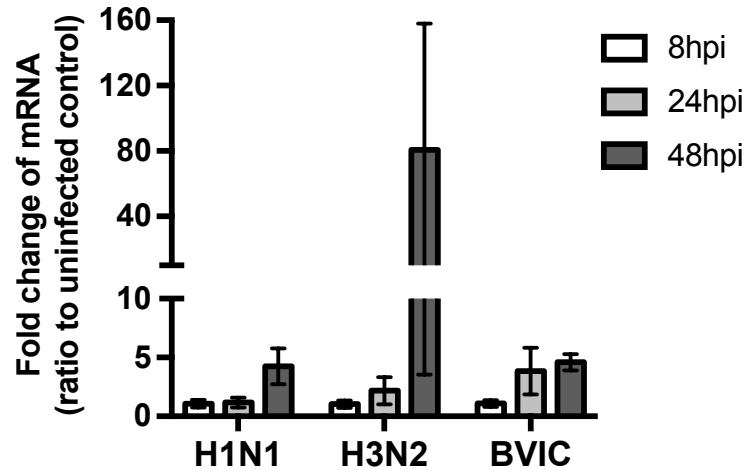

Supplement: Supplementary file 3 — Figure S1. Expression of MUC15, MUC13 and MUC3A in seasonal influenza H1N1, H3N2 and B infection. Quantitative real-time RT-PCR analysis of MUC15, MUC13 and MUC3A gene mRNA expression in hNECs infected with seasonal influenza H1N1, H3N2, and B, with uninfected control subjects as baseline (n = 4) and using PGK1 as the internal control. Relative mRNA expression levels were calculated using 2^ (−ΔΔCt) method; the fold change was calculated as fold increases from uninfected control subjects. * and ** denotes P value of less than 0.05 and 0.01 compared with uninfected control, respectively. (PDF 37 kb) [file 12879_2019_4213_MOESM3_ESM.pdf]

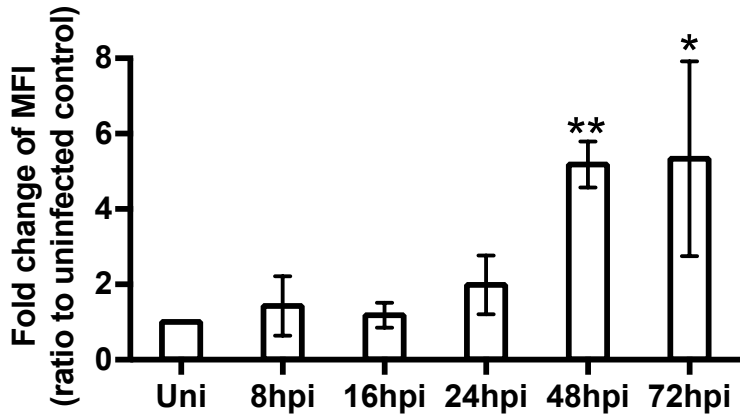

Supplement: Supplementary file 4 — Figure S2. Mean fluorescence intensity (MFI) of MUC15 in infected hNECs. Five different images were captured from every slide and the mean immunofluorescence intensities (MFI) of MUC15 were measured using Image J (n = 5). Data was then normalized to each uninfected control. *, **, ***, **** denotes P value of less than 0.05, 0.01, 0.001, < 0.0001 compared with uninfected control, respectively. Median values with 25th and 75th percentiles are indicated by error bar. Uni: uninfected control. (PDF 21 kb) [file 12879_2019_4213_MOESM4_ESM.pdf]

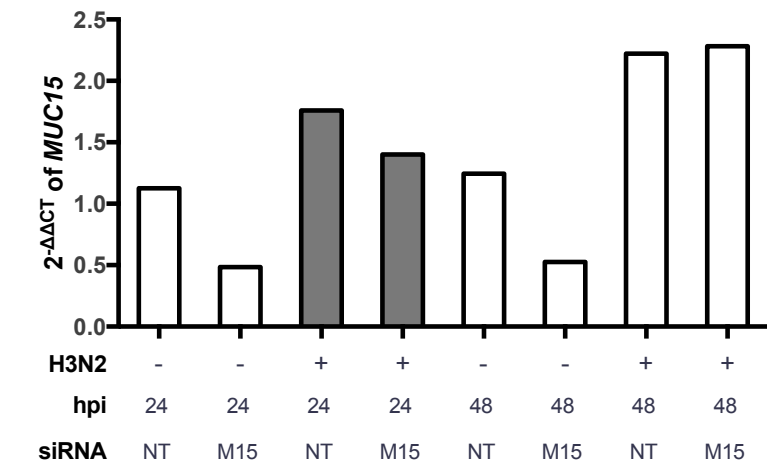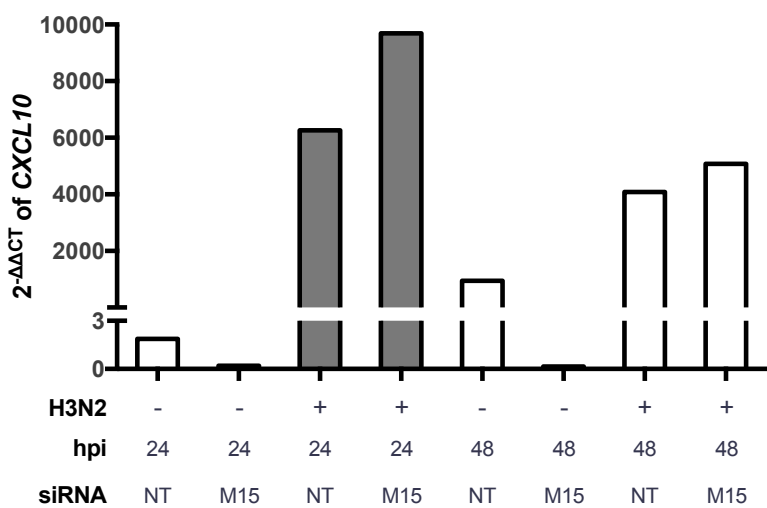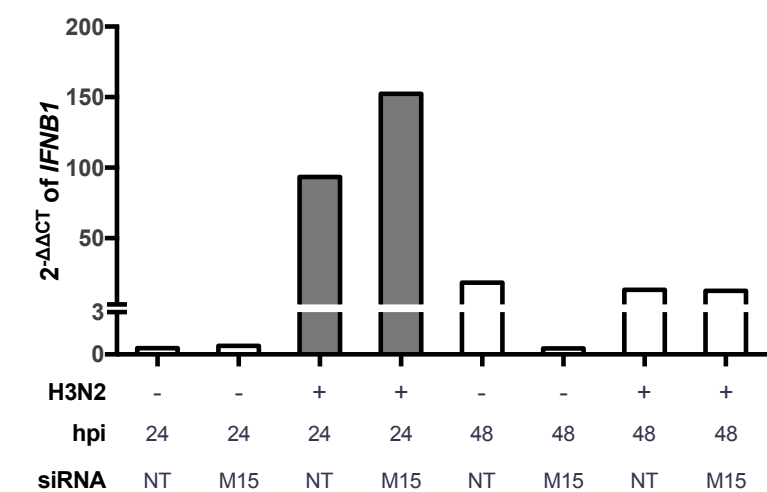

Supplement: Supplementary file 5 — Figure S3. Expression of MUC15, IFNβ and CXCL10 in MUC15 siRNA knockdown in hNECs. siRNA knockdown of MUC15 in hNECs (n = 1). siRNA knockdown of MUC15 was achieved stronger in non-infected hNECs (both 24 and 48 hpi). Conversely, the knockdown was greatly reduced in infected hNECs when MUC15 is highly induced; and the knockdown is only slightly achieved at 24 hpi (grey bars). Quantitative real-time RT-PCR analysis of MUC15, IFNβ and CXCL10 was performed using PGK1 as internal control. Relative mRNA expression levels were calculated using 2^ (−ΔΔCt) method. Hpi: hours post infection; NT: non-targeting control; M15: MUC15 siRNA. (PDF 41 kb) [file 12879_2019_4213_MOESM5_ESM.pdf]
